# Exon-skipping antisense oligonucleotides targeting SUMO1 and SUMO2 demonstrate chemosensitizing effects in NSCLC cells in tissue culture

**DOI:** 10.21203/rs.3.rs-9361591/v1

**Published:** 2026-05-05

**Authors:** Andrea Garcia-Morin, Rebeca Orozco-Sepulveda, Yesenia Juarez-Vargas, Claudia Banuelos, Isabel Gutierrez-Zubiate, German Rosas-Acosta

**Affiliations:** The University of Texas at El Paso; The University of Texas at El Paso; The University of Texas at El Paso; The University of Texas at El Paso; The University of Texas at El Paso; The University of Texas at El Paso

## Abstract

Lung cancer remains a leading cause of cancer-related death, highlighting the urgent need for new treatment strategies. SUMOylation, a post-translational modification that regulates DNA repair, replication, and cell cycle progression, is often increased in cancers and is a promising therapeutic target. Previously, we identified alternative splicing events affecting the SUMO transcripts, resulting in variant mRNAs coding for both non-conjugatable (SUMO1α and SUMO2α) and conjugatable (SUMO3α) isoforms—potentially helping to regulate overall cellular SUMOylation. In this study, we investigated whether increasing the levels of the transcripts coding for the non-conjugatable isoforms could decrease SUMOylation and improve chemotherapy efficacy in non-small cell lung cancer (NSCLC). To test this, we designed two sets of exon-skipping morpholinos called SUM2IN and SUMO1IN, targeting the pre-mRNAs of SUMO2 and SUMO1, respectively, and tested them in A549 and HCC827 cell lines. While treatment with SUM2IN significantly increased SUMO2α mRNA and reduced overall SUMO1 and SUMO2 conjugation in all tested cell lines, SUM1IN affected the proportion of SUMO1 mRNA variants and overall SUMO conjugation in a cell-type-specific manner. Still, both SUM2IN and SUM1IN altered cell cycle progression, decreased cell growth, and enhanced the cytotoxic effects of cisplatin and etoposide in A549 and HCC827 cells, while having a notably smaller impact on these parameters in the non-malignant lung fibroblast cell line HEL-299. Therefore, SUM2IN and SUM1IN display chemosensitizing activity against NSCLC and provide a strong foundation for developing SUMO-targeted chemosensitizers for NSCLC and other aggressive human cancers.

## Introduction

Lung cancer remains the leading cause of cancer-related mortality, with non-small cell lung cancer (NSCLC) comprising 80% of cases [1] and a dismal 32% five-year survival rate (2012–2018) [2]. Standard chemotherapy often fails due to the rapid development of resistance, underscoring the need for strategies that exploit tumor-specific vulnerabilities, such as dysregulated post-translational modifications, to improve chemotherapeutic outcomes [3, 4].

SUMOylation, the covalent attachment of Small Ubiquitin-like MOdifier (SUMO) proteins to targets, drives cancer hallmarks including unregulated proliferation [5], apoptosis resistance [6], and cytoskeletal remodeling essential for invasion and metastasis [7]. Unlike ubiquitination, which primarily targets proteins for degradation [8], SUMOylation fine-tunes protein function in a protein-specific manner, affecting cellular processes as diverse as nucleocytoplasmic transport [9], transcription [10, 11], DNA repair [12, 13], and cell cycle control [14, 15]. In NSCLC, numerous components of the SUMO pathway, including the SUMO proteins themselves, the essential E1 and E2 enzymes [16], and the optional E3 ligases, are upregulated, correlating with malignancy [17, 18]. Thus, inhibiting SUMOylation limits tumor growth, suggesting therapeutic potential [19, 20].

Humans express five SUMO paralogs (SUMO1–5), each encoded by a separate gene. SUMO1 shares ~ 45% identity with SUMO2/3, and SUMO2 and SUMO3 are 95% identical to each other [21]. SUMO4 and SUMO5 are poorly expressed and tissue-restricted [22, 23]. All SUMOs feature conserved ubiquitin-like domains and C-terminal diglycine motifs [24, 25], which provide the carboxyl-end group used for conjugation via a well-conserved pathway involving the required E1 activating and E2 conjugating enzymes (SAE1/2 and UBC9, respectively), and the optional E3 ligases [22, 26].

In our previous study, we reported how alternative splicing generates different isoforms of the three main SUMO paralogs. SUMO1 produces three mRNA variants coding for two SUMO isoforms, while SUMO2 and SUMO3 each produce two mRNA variants coding for two isoforms [27]. For all three SUMO genes, the most abundant SUMO transcript is the normally spliced one, coding for the prototypical SUMO paralog protein. The alternatively spliced forms, coding for what we named the SUMO alphas, were substantially less abundant [27]. Importantly, in all cell types studied, including A549 cells (a well-known NSCLC cell line), SUMO2 transcripts were by far the most abundant transcripts [27], emphasizing the relevance of SUMO2 for cellular function. As for the SUMO alphas, SUMO1α and SUMO2α proved to be non-conjugatable, implying that the alternative splicing of the SUMO transcripts may help regulate cellular SUMOylation by reducing the functional pool of SUMO proteins and global conjugation [27], a fact that could be used to produce a vulnerability in SUMO-dependent tumors. Thus, we hypothesized that increasing the expression of the non-conjugatable SUMO2α and SUMO1α via splicing modulation would downregulate global SUMOylation, thereby enhancing chemotherapy cytotoxicity in NSCLC while sparing normal lung cells, as exemplified by HEL299.

Here, we tested this hypothesis in A549, HCC827, and HEL299 cells by employing sets of vivo-morpholinos targeting splicing donor and acceptor sites at exon-intron junctions, thereby driving exon-skipping events that increased the production of the SUMO alphas. The effects mediated by these events were subsequently assessed by measuring their impact on proliferation, migration, global SUMOylation, and potential synergy with cisplatin and etoposide.

We observed the expected tumor-selective SUMO downregulation, which in turn enhanced cell death, lowered chemoresistance, and decreased cell migration. Our findings lay the groundwork for a new method to increase tumor-cell chemosensitivity by modulating cellular SUMOylation through boosting naturally occurring low-frequency alternative splicing events. Given that morpholino-based therapeutics have been approved since 2016 [28], the approach presented here could be easily moved into clinical trials.

## Materials & Methods

### 1. Vivo-Morpholino Design:

To induce specific exon skipping events resulting in the production of the desired transcript variants, we designed pairs of SUMO-specific vivo-morpholinos targeting the intron-exon splice-acceptor and exon-intron splice-donors sites surrounding the exon to be skipped. We designed the vivo-morpholinos following well-established principles of targeted mRNA splicing modulation, as follows: The vivo-morpholinos were designed to be fully complementary to the target mRNA sequence. Each vivo-morpholino was exactly 25 nucleotides (nt) in length, with a guanine-cytosine (GC) content ranging between 40 to 60% to optimize stability and hybridization efficiency [29]. Secondary structures and self-complementarity within the morpholino sequences were minimized to reduce potential steric hindrance and ensure efficient binding. Additionally, complementarity between 5’ and 3’ terminal end sequences was also avoided to prevent potentially pro-thrombotic effects during future clinical implementation [30]. The sequences of the vivo-morpholinos used, and their target sequences, are indicated in Table 1. The sequence of the vivo-morpholinos targeting SUMO2 and the methodology for their implementation in tissue culture were previously described in detail by our group [31].

### 2. Cell and Tissue Culture:

A549, HCC827, and HEL299 cells were from ATCC (American Type Culture Collection). All cells were grown at 37°C, 5% CO_2_, in 1x Complete Medium consisting of 1x DMEM containing high glucose, pyruvate, and GlutaMAX^™^ (Gibco^™^, ThermoFisher Scientific, Inc.), supplemented with 10% Fetal Bovine Serum.

### 3. Vivo-Morpholino Treatment:

Vivo-morpholinos were resuspended in sterile distilled water at an initial concentration of 400 μM and kept at room temperature in a dark container. For the vivo-morpholino treatment, cells were plated at densities tailored to each experiment (see protocols below for specific cell densities in cells/mm^2^) and incubated overnight under standard conditions to allow attachment and growth. The following day, vivo-morpholino mixes were prepared by diluting them in Opti-MEM^™^ I Reduced-Serum Medium (Gibco^™^, ThermoFisher Scientific, Inc.) to a final concentration of 4 μM (working solutions). Vivo-morpholino treatments were performed by aspirating the existing culture medium, immediately adding the designated working solutions, and incubating the cells for 4 hours at 37°C and 5% CO_2_. Subsequently, 3 volumes of 1x Complete Medium were added to each well, thereby bringing the vivo-morpholinos concentration down to 1 μM. The cells were further incubated for 24 hours under the same conditions before subsequent analyses.

### 4. RNA Purification:

For RNA purification, A549, HCC827, or HEL299 cells were plated at a density of 5 × 10^5^ cells/well in 6-well plates, treated as described above under “Vivo-Morpholino Treatment,” and cultured for 24h at 37°C with 5% CO_2_. Cells were collected by aspirating the culture medium, gently washing with 1 mL of 1× PBS, and lysing directly in the wells with 250 μL of Buffer RLT. Total RNA was purified using the RNeasy Mini Kit^®^ in combination with the QIAshredder^®^ system (QIAGEN, Inc., Redwood City, CA), according to the manufacturer’s instructions. Purified RNA was eluted in 40 μL of RNase-free Milli-Q water and stored at −80°C until further use.

### 5. Assessment of Purified RNA’s Quality and Quantity:

RNA concentration and purity were determined using a NanoDrop^™^ One/OneC Microvolume UV–Vis spectrophotometer (ThermoFisher Scientific, Inc.) following the manufacturer’s protocol. RNA integrity was assessed by formaldehyde–agarose gel electrophoresis. Samples were considered suitable for downstream analyses when distinct 28S and 18S rRNA bands were observed, with the 28S band exhibiting approximately twice the intensity of the 18S band, and when high-molecular-weight RNA species were clearly visible up beyond the 10 kbp marker.

### 6. RT-qPCR:

Reverse transcription quantitative PCR (RT–qPCR) was performed using the iTaq^™^ Universal SYBR^®^ Green One-Step Kit (Bio-Rad Laboratories, Inc., Hercules, CA) according to the manufacturer’s instructions. Briefly, 100 ng of total RNA was combined with 10 μL of reaction mix, 2 μL of forward primer, 2 μL of reverse primer, 0.25 μL of iScript^™^ reverse transcriptase, and nuclease-free water to a final volume of 20 μL. Reactions were performed in triplicate for each sample, and negative controls lacking RNA template were included in all experiments.

Amplification was carried out using a MyGo Pro Real-Time PCR thermocycler (Azura Genomics, Inc., Raynham, MA) with MyGo software (Mac OS X platform). The thermal cycling conditions were as follows: reverse transcription at 50°C for 10 min, initial denaturation at 95°C for 3 min, followed by 40 cycles of denaturation at 95°C for 10s (ramp rate 5°C/s) and annealing/extension at 60°C for 30s (ramp rate 4°C/s). To confirm amplification specificity, a high-resolution melting curve analysis was subsequently performed with an initial step at 60°C for 1 min, followed by a gradual increase in temperature at 0.05°C/s to 95°C. The RT–qPCR products were additionally analyzed by electrophoresis on 1.5% agarose gels using 5 μL of each reaction.

### 7. Immunoblot Analyses:

For immunoblot analyses, A549, HCC827, and HEL299 cells were plated at a density of 5 × 10^5^ cells/well in 6-well plates and allowed to adhere and grow for 24h at 37°C and 5% CO_2_. Cells were treated as described above in “Vivo-Morpholino Treatment” and collected by aspirating the culture medium using vacuum suction, washing gently with 1× PBS for about 1 min, aspirating the 1× PBS, and adding 250 μL of boiling 4× Sample Buffer (200 mM Tris-HCl/pH 6.8/, 8% SDS, 40% glycerol, and 0.04% bromophenol blue) directly to each well. The resulting lysates were transferred to 1.5 mL microcentrifuge tubes and passed through a 29-gauge insulin syringe 7–10 times to shear genomic DNA and reduce viscosity. Samples were supplemented with β-mercaptoethanol to a final concentration of 5% and boiled for 5 min before electrophoresis.

For SDS–PAGE, 15 μL of each sample and 5 μL of molecular weight standards were loaded onto 14cm × 12cm × 0.15cm discontinuous 10% SDS–polyacrylamide gels and electrophoresed at 90 V for 30 min, followed by 120 V for approximately 1h, or until the dye front reached the bottom of the gel on a Hoeffer^™^ SE 600 Series Vertical Electrophoresis System (Fisher Scientific, Thermofisher Scientific, Inc.). Following electrophoresis, gels were equilibrated in transfer buffer (20% Methanol, 25 mM Tris, 192 mM Glycine, pH 8.3) for 10 min at room temperature. Proteins were transferred to PVDF membranes using a wet transfer system at a constant current of 1.6 mA for 110 min using an Owl^™^ VEP-3 Large Tank Electroblotting System (ThermoFisher Scientific, Inc.). Membranes were activated in 100% methanol before transfer and equilibrated in transfer buffer.

Upon transfer, PVDF membranes were air-dried overnight, rehydrated in methanol for less than 1 min, washed three times with distilled water, and washed twice with 1× PBS. Membranes were then blocked with blocking solution (3% fat-free milk in 1× PBS + 0.1% Tween-20) for 1 h at room temperature. Primary antibodies were added directly to the blocking solution and incubated overnight at 4°C with gentle rocking.

Following primary antibody incubation, membranes were washed three times with 1× TPBS (1× PBS + 0.1% Tween-20) for 3 min each and incubated with secondary antibodies (1:5000 dilution in blocking solution) for 1h at room temperature. Membranes were subsequently washed three times with 1× TPBS and once with 1× PBS.

For detection, membranes were incubated with enhanced chemiluminescence substrate prepared according to the manufacturer’s instructions, and signals were visualized using an iBright imaging system. For loading control validation, membranes were reprobed with anti-GAPDH antibodies using the same immunoblotting procedure, starting at the blocking step.

The primary and secondary antibodies used were as follows:

SUMO1: Given the limitations inherent to the use of single antibodies in the detection of SUMO1 conjugates [32], a cocktail of three different antibodies was used: rabbit polyclonal anti-SUMO1 Y299 from Abcam (Abcam, Cambridge, UK), 1:5,000 dilution; rabbit monoclonal anti-SUMO 1 C9H1 from Cell Signaling (Cell Signaling Technology, Inc., Danvers, MA), 1:5,000 dilution; and, a previously reported rabbit polyclonal serum against SUMO-1 developed in house (#12783), 1:1,000 dilution [33].

SUMO2/3: Rabbit polyclonal anti-SUMO2 (Sentrin 2) from Zymed (51–9100)(Zymed Technologies, ThermoFisher Scientific, Inc.), 1:3,000 dilution.

UBC9: Rabbit monoclonal anti-UBE21/UBC9 [EP2938Y] from Abcam (ab75854)

GAPDH: Rabbit monoclonal anti-GAPDH (14C10), from Cell Signaling (Cell Signaling Technology, Inc.), 1:5,000 dilution.

Secondary anti-rabbit: Mouse anti-rabbit IgG-HRP conjugated (sc-2357), from Santa Cruz Biotech (Santa Cruz Biotechnology, Inc., Dallas, TX), 1:5,000 dilution.

### 8. Cytotoxicity Assay:

Cytotoxicity was assessed using a differential nuclear staining assay as previously reported [34]. Briefly, A549, HCC827, and HEL299 cells were plated at a density of 4 × 10^3^ cells/well in 100 μL of culture medium in a 96-well plate. The cells were incubated at 37°C in 5% CO_2_ for 24 hours to ensure proper attachment and growth before treatment. Following incubation, cisplatin or etoposide was used at a concentration 4 times lower than their cell line-specific EC50. SUM2IN, SUM1N, and the Scrambled control morpholinos were used at the same concentration as stated above. 0.1% DMSO and acid water (pH 6) were used as vehicle controls. 50 μM H_2_O_2_ was used as a positive control for cell killing. Following the 72-hour incubation period, cells were stained for analysis using 10μL per well of a staining solution containing 940 μL 1×PBS, 40 μL Hoechst stain, and 20 μL Propidium Iodide (PI). After incubation with the stain for 1 hour at 37°C, fluorescence imaging and viability analysis were conducted using the ImageXpress Pico | ImageXpress^®^ Pico system (Molecular Devices).

### 9. Cell Migration Assay:

A549 cells were plated at a density of 1.3 × 10^5^ cells/well in 1 mL of media in a 12-well plate and incubated at 37°C for 24 hours to allow adherence and growth. Once the wells reached 90% confluence, a scratch assay was performed as follows: The culture medium was aspirated from each well, and 1 mL of 1× PBS was added for washing. A 200 μL micropipette tip was used to create a vertical scratch (8 scratches per well), ensuring a consistent force and speed was applied throughout the scratching process. Wells were then washed three times with 1mL of 1× PBS to remove all the detached cells. Morpholino treatments were added to the respective wells, and images of the initial wound area (Time 0, T0) were captured using confocal microscopy. Cells were incubated at 37°C for 48 hours, with additional images recorded at 24 hours (T24) and 48 hours (T48) to monitor wound closure over time.

### 10. Cell Cycle Analysis:

Cells were seeded at a density of 3.5 × 10^4^ cells/well for A549 cells or 7 × 10^4^ cells/well for HCC827 cells, using 1 mL of complete medium per well in a 24-well plate. The cells were incubated at 37°C in 5% CO_2_ for 24 hours to ensure proper attachment before treatment. Following incubation, the culture medium was aspirated, and cells were treated with vivo-morpholinos as stated above. For cells treated with the vivo-morpholinos (SUM2IN, SUM1IN, and Scr) in combination with either cisplatin or etoposide, a dose 10 times lower than the EC50 was used for the chemotherapeutic agent. 0.1% DMSO and acid water (pH 6) were used as vehicle controls, and camptothecin at 10 μM was used as a positive control. Upon addition of the treatment, cells were incubated for an additional 48 hours at 37°C with 5% CO_2_, and subsequently collected for analysis. In preparation for cell cycle analysis by flow cytometry, a NIM-DAPI Staining Solution was prepared by adding 0.6% NP-40 to 1× PBS containing 1% DAPI. Then, conditioned medium from each well was collected and transferred into a 5 mL tube. 250 μL of trypsin was added to each well, and the plate was incubated for 10 minutes at 37°C to detach the cells. Detached cells were collected and combined with the previously collected media in the 5 mL tube to retain both adherent and non-adherent populations. The samples were centrifuged at 1,200 rpm for 5 minutes. The supernatant was decanted, and the cell pellet was resuspended in 200 μL of NIM-DAPI solution and 100 μL of 1×PBS. The mixture was vortexed briefly to ensure uniform staining. Finally, each sample was read using the Beckman Coulter Gallios Flow Cytometer (Beckman Coulter Life Sciences).

### 11. Colony Formation Assay:

Cells were plated at a density of 2 × 10^3^ cells/well for A549 cells or 3 × 10^3^ cells/well for HCC827 on a 12-well plate and cultured for 24h at 37°C, 5% CO_2_ to allow proper cell attachment and growth. Following incubation, the cells were treated with the specific vivo-morpholino (SUM2IN, SUMI1N, and Scr) for 24 h. After 24 h, the cells were treated with either cisplatin or etoposide for 3 consecutive days, with freshly prepared chemotherapeutic agents added daily. On the 4th day, the treatment was removed, and complete medium was added to the wells. Cells were then incubated for an additional 5 days, with the medium changed as necessary. Following the incubation period, colonies were fixed and stained using 0.5% crystal violet solution. The stained colonies in each well were counted and analyzed using ImageJ software. Images of stained colonies were captured for quantification.

### 12. Statistical Analyses and Ethical Statement:

Copy number estimates (CNest) were calculated using calibration curves generated as described in our previous paper [27], based on average Cq values obtained from triplicate experiments, each measured in triplicate RT–qPCR reactions. All statistical analyses were performed using GraphPad Prism (v.6.0). All experimental procedures were conducted in accordance with institutional guidelines for biological safety and recombinant DNA research, as approved by the Institutional Biosafety Committee (IBC), Institutional Review Board (IRB), and Environmental Health and Safety (EH&S) Department at The University of Texas at El Paso.

## Results

### Antisense Oligonucleotides Targeting Splicing Signals in the SUMO2 and SUMO1 pre-mRNAs Trigger Cell-Type Specific Effects

Numerous studies have shown that SUMOylation is often elevated in various types of cancer, and the extent of these increases seems proportional to the prognosis [35]. As a result, targeting the SUMOylation system has been explored as a promising anticancer treatment, leading to the development of several SUMO inhibitors, one of which (TAK-981) reached clinical trials [36]. In our previous research, we (i) described the alternative splicing of transcripts coding for the main SUMO paralogs produced in humans, i.e., SUMO1, SUMO2, and SUMO3; (ii) confirmed that SUMO2 is by far the most abundantly expressed SUMO gene; and (iii) identified alternatively spliced transcripts that produce non-conjugatable isoforms of SUMO1 and SUMO2, called SUMO1α and SUMO2α, respectively [27].

**Figure 1.**
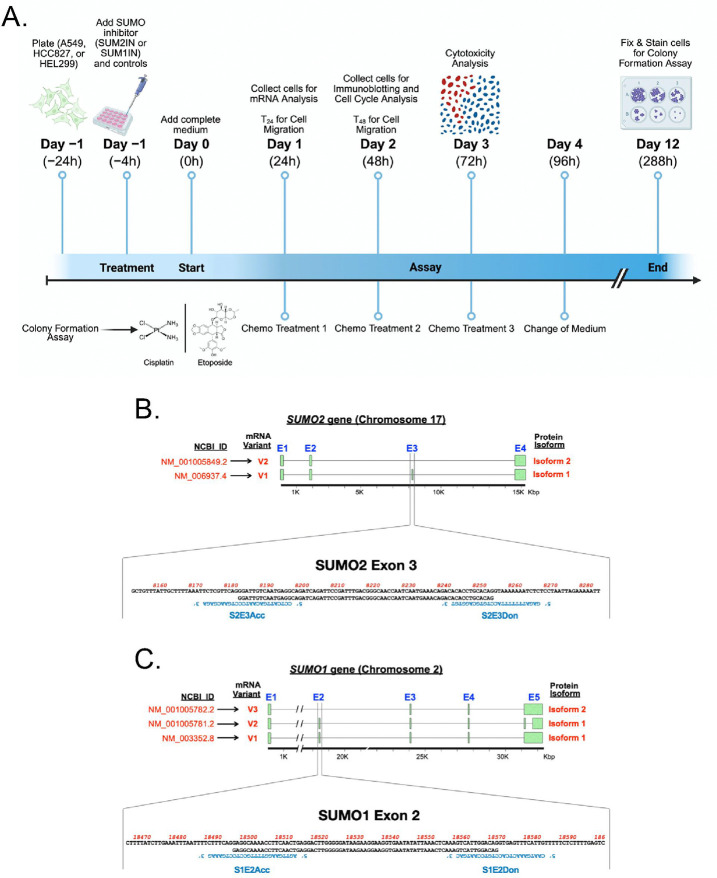
Experimental design, workflow, and vivo-morpholino designs for this study. **(A)** Timeline illustrating key time points for treatment administration and sampling for downstream assays, including mRNA analysis by RT-qPCR, protein level assessment by Immuniblotting, cytotoxicity analysis by Differential Nuclear Staining, cell cycle analysis by Flow Cytometry, cell migration by Wound Healing Assay, and long-term survival by Colony Formation Assay. **(B & C)** Schematics of the SUMO2 and SUMO1 genes, exon distribution, and targets for SUM2IN and SUM1IN. The nucleotide sequences of the exons targeted for exon-skipping (Exon 3 for SUMO2, and Exon 2 for SUMO1) are shown. The top sequence represents the pre-mRNA, whereas the bottom sequence represents the exons. The sequences of the morpholino pairs that constitute SUM2IN (S2E3Acc + S2E3Don) and SUM1IN (S1E2Acc + S1E2Don) are indicated in blue. Treatment with SUM2IN results in the skipping of Exon 3, triggering an increase in S2V2, coding for SUMO2α. Treatment with SUM1IN results in the skipping of Exon 2, triggering an increase in S1V3, coding for SUMO1α.

For SUMO2, the normally spliced variant, SUMO2 variant 1 (S2V1), accounts for over 98% of all transcripts produced. However, since the main alternatively spliced form of the SUMO2 transcript, SUMO2 variant 2 (S2V2), encodes a non-conjugatable SUMO isoform (SUMO2α), we hypothesized that increasing the proportion of S2V2 relative to S2V1 could significantly reduce overall protein SUMOylation, likely mimicking the effect of inhibiting the enzymatic components of the SUMOylation system. To switch between S2V1 and S2V2 production, we aimed to induce exon skipping during SUMO2 pre-mRNA splicing. For this, we designed SUM2IN, a mix of two vivo-morpholinos (S2E3Acc and S2E3Don) targeting the splicing acceptor and donor sites surrounding exon 3 in the SUMO2 pre-mRNA (Fig. 1B).

**Figure 2.**
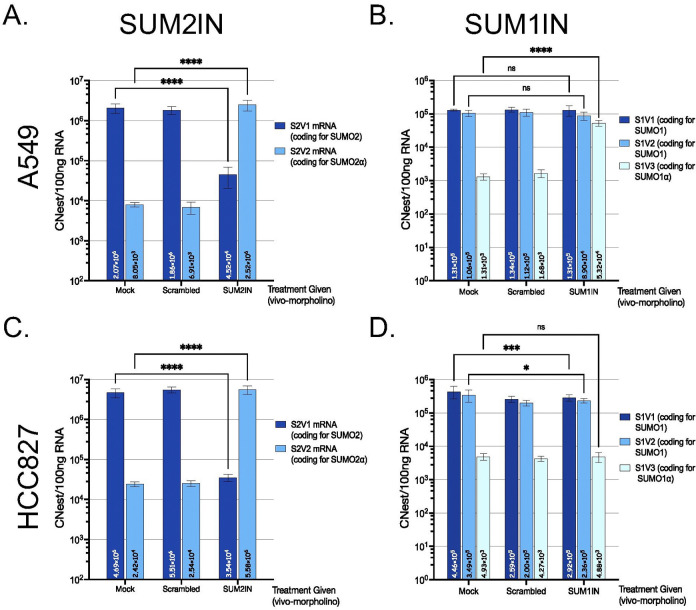
Changes in the abundance of the main SUMO2 and SUMO1 mRNA variants triggered by SUM2IN and SUM1IN, respectively, in A549 and HCC827 cells. A549 and HCC827 cells were treated with either, Opti-MEM I only (Mock), a scrambled morpholino (scrambled), SUM2IN, or SUM1IN, as indicated. At 24h post-treatment, cells were collected, RNA was purified, and the copy number estimate per 100ng of RNA (Cnest/100ng RNA) for the variants transcripts were calculated by RT-qPCR. **(A and C)** Changes triggered by SUM2IN treatment in A549 and HCC827 cells, respectively. **(B and D)** Changes triggered by SUM1IN treatment in A549 and HCC827 cells, respectively. SUM2IN specifically affects the splicing of the SUMO2 pre-mRNA in both A549 and HCC827 cells, triggering a near inversion in the abundance of S2V2 and S2V1 in HCC827 cells, and a 172x increase in S2V2 and a 45x decrease in S2V1 in A540. SUM1IN affects the splicing of the SUMO1 pre-mRNA, triggering an increase in S1V3 in A549 cells, but has no apparent effect in HCC827 cells. Each bar represents the mean ± SD of three independent experiments, each performed in triplicate.

**Figure 3.**
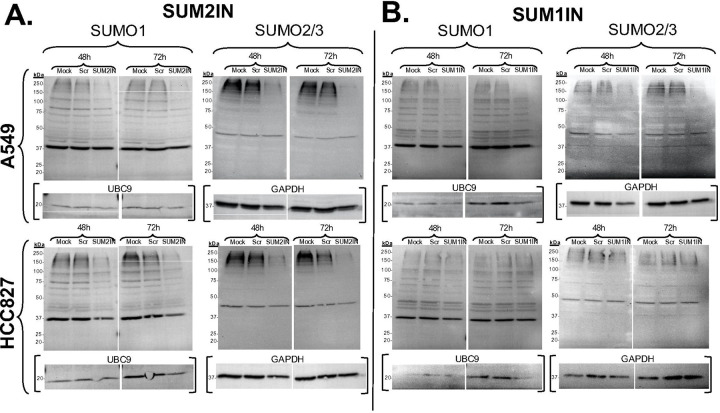
Changes in global SUMO1 and SUMO2/3 SUMOylation triggered by treatment with SUM2IN and SUM1IN in A549 and HCC827 cells, as measured by immunoblotting. Cells were collected at 48 or 72 hours post-treatment with either medium alone (Mock), scrambled morpholino control (Scr), SUM2IN, or SUM1IN. Membranes were sequentially probed with antibodies to SUMO1, SUMO2/3, UBC9, and GAPDH. **(A)** Effects mediated by SUM2IN treatment. SUM2IN reduces SUMO2 SUMOylation by 48 hours, and makes it almost undetectable by 72 hours. SUM2IN also decreases SUMO1 SUMOylation at both 48 and 72 hours, indicating that SUM2IN treatment broadly suppresses global SUMOylation. **(B)** Effects mediated by SUM1IN treatment. In A549 cells, SUM1IN decreases SUMO1 and SUMO2 SUMOylation at both 48 and 72 hours. In contrast, SUM1IN does not affect SUMO1 or SUMO2 in HCC827 cells. The data presented is representative of results obtained in at least three independent experiments.

To assess how effectively SUM2IN influences the splicing of SUMO2 and thereby increases the abundance of S1V2, we conducted RT-qPCR analyses. In both A549 and HCC827 cells, SUM2IN efficiently promoted exon 3 skipping, leading to an increase in S2V2 production and a decrease in S2V1 levels (Fig. 2A&C). To investigate how these changes in SUMO2 variant levels impacted overall SUMOylation, we performed immunoblot analyses for SUMO1 and SUMO2/3. Similar trends were observed in both A549 and HCC827 cells following SUM2IN treatment. Specifically, SUM2IN substantially reduced SUMO2/3 protein levels by 48 hours, with SUMO2/3 SUMOylation becoming nearly undetectable by 72 hours. Importantly, SUM2IN also lowered SUMO1 conjugation, indicating a broad suppression of global SUMOylation (Fig. 3A).

Since SUM2IN successfully triggered the expected exon-skipping event, we aimed to use a similar method to increase the expression of SUMO1α, encoded by S1V3. Therefore, we created SUM1IN, which includes two vivo-morpholinos (S1E2Acc and S1E2Don) that target the splicing acceptor and donor sites flanking exon 2 of the SUMO1 pre-mRNA, respectively (Fig. 1C). We chose SUMO1 over SUMO3 because (i) SUMO1 is more abundant; (ii) the splicing process of SUMO1 closely resembles that of SUMO2, involving an exon skipping event that produces SUMO1 variant 3 (S1V3), the mRNA coding for the non-conjugatable SUMO1 isoform (SUMO1α); and (iii) the main alternative splicing event affecting SUMO3 transcripts results in an exon extension that produces an mRNA coding for a conjugatable protein, SUMO3α. We therefore hypothesized that selectively increasing S1V3 production while reducing S1V1 would significantly increase SUMO1α, leading to a global decrease in SUMOylation, similar to the effect observed with SUM2IN.

To evaluate the efficacy of SUM1IN, we used the same experimental setup as for SUM2IN. To determine if SUM1IN affected SUMO1 splicing and increased the relative abundance of the SUMO1α-coding isoform (S1V3), we performed RT-qPCR analyses. In A549 cells, SUM1IN effectively caused exon 2 skipping, resulting in higher levels of the S1V3 transcript, but unexpectedly, it did not change the levels of S1V1 and S1V2 transcripts (Fig. 2B). In HCC827 cells, SUM1IN failed to increase S1V3 expression and did not significantly affect S1V1 or S1V2 transcripts (Fig. 2D). To see if SUM1IN treatment affected overall SUMOylation, we carried out immunoblot analyses for SUMO1 and SUMO2/3. Cell-type–specific effects were observed after SUM1IN treatment. In A549 cells, SUM1IN significantly reduced SUMO1 protein levels by 48 hours, and by 72 hours, SUMO1-SUMOyation was nearly undetectable, while also decreasing SUMO2/3 conjugation, indicating broad suppression of SUMOylation, similar to the effects seen with SUM2IN. In contrast, SUM1IN treatment did not change SUMO1 or SUMO2/3 protein conjugation in HCC827 cells (Fig. 3B). Overall, these results show that SUM1IN’s effects on SUMOylation depend on the cell type and may not be directly linked to triggering exon-skipping events.

### SUM2IN and SUM1IN Promote Apoptosis and Disrupt Cell Cycle Progression and Cell Migration in NSCLC Cells

**Figure 4.**
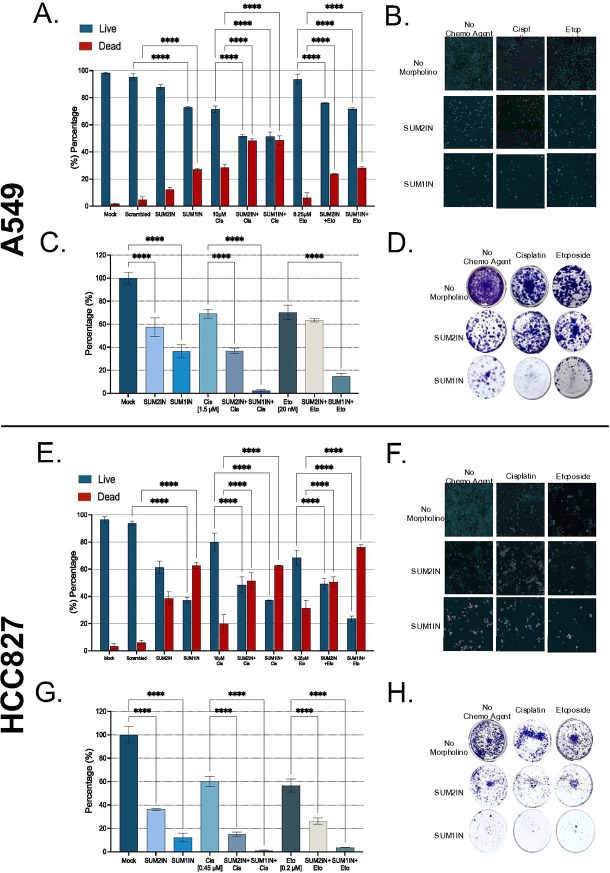
Treatment with SUM2IN and SUM1IN increases the killing effect mediated by cisplatin and etoposide as measured by a Differential Nuclear Staining Assay and a Colony Formation Assay. **(A and B)** A549 cells plated at 4,000 cells/well in 96-well plate were treated with either SUM2IN, SUM1IN, cisplatin, and etoposide, alone or in combination as indicated. 96h post-treatment, the cells were stained with Hoechst and Propidium lodide, and read using a 1xPico Bioimager MolDev. Panel A shows data representing the average values obtained from six different wells per concentration pear each treatment, and summarizes the averages and standard deviations from 3 independent experiments. Panel B shows representative images captured by the 1xPico Bioimager. **(C and D)** A549 cells were seeded at 2,000 cells/well in a 6-well plate and allowed to attach overnight in a humidified incubator at 37°C. Next morning, the cells were treated with SUM2IN or scrambled morpholinos at 4μM. The next day, the plates were treated with daily additions of cisplatin (1.5μM or 0.45μM) or etopoiside (20nM or 0.2μM) for 3 consecutive days, as represented in Figure 1. Then, the plates were incubated for 5 additional days at 37°C and 5% CO_2_. Subsequently, each well was fixed by staining with a 0.5% crystal violet solution. The colonies stained in each well were counted and photographed with ImageJ software. Panel C shows data representing the average values obtained from four different wells per concentrtion prear treatment, and summarized the averages and standard deviations from 2 independent experiments. Panel D shows representaive images of the stained colonies. **(E-H)** The same procedures described in (A–D) were performed using HCC827 cells. In both cell lines, treatment with cisplatin or etoposide in combination with either SUM2IN or SUM1IN consistently increased cytotoxicity compared to drug treatment alone.

Given the dependence of tumor cells on an active SUMOylation system and the observed reduction in overall SUMOylation caused by SUM2IN and, to some extent, SUM1IN, we then decided to assess their anti-tumor activity. To do this, we performed cytotoxicity assays using a differential nuclear staining method and clonogenic survival tests with a colony formation assay (Fig. 1A). The cytotoxicity results showed that altering SUMO2 and SUMO1 levels significantly decreased the number of viable cells (Fig. 4A, B, E & F). Consistent with these results, the colony formation assays indicated a substantial reduction in colony numbers following treatment with SUM2IN and SUM1IN in both A549 and HCC827 cells (Fig. 4C, D, G & H).

In A549 cells, 24-hour exposure to SUM2IN increased cell death by 12%, whereas similar exposure to SUM1IN increased it by 27% (Fig. 4A). Long-term exposure (15 days) led to about a 40% reduction in colony formation with SUM2IN and 60% with SUM1IN (Fig. 4C). In HCC827 cells, short-term treatment with SUM2IN increased cell death by 38%, while SUM1IN increased it by 62% (Fig. 4E). Likewise, long-term treatment decreased colony numbers by nearly 60% with SUM2IN and 85% with SUM1IN (Fig. 4G), indicating that SUM1IN is considerably more cytotoxic than SUM2IN in both cell lines. Overall, these results show that SUM2IN and SUM1IN effectively disrupt vital processes in NSCLC, leading to cell death.

**Figure 5.**
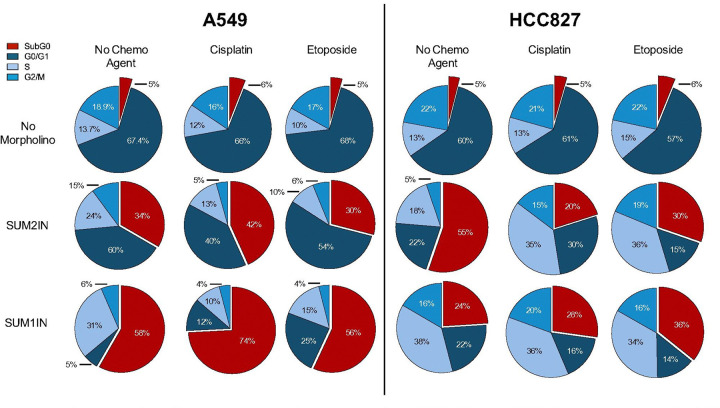
Changes in cell-cycle progression triggered by SUM2IN and SUM1IN treatment. A549 (left) and HCC827 (right) cells were treated with the indicated combination of morpholinos and chemotherapeutic agents. At 48 hours post-treatment, the cells were stained with NIM-DAPI and analyzed by flow cytometry. Camptothecin and scrambled morpholinos were used as controls (see Supplementary Fig. 2). SUM2IN and SUM1IN were used at a final concentration of 4 μM. Etoposide and cisplatin were used at final concentrations of 200 nM and 1 μM in A549 cells, and 0.2 μM and 0.45 μM in HCC827 cells, respectively. In A549 cells, SUM2IN decreased the proportion of cells in G0/G1 and increased the SubG0 population, indicating enhanced cell death. In HCC827 cells, SUM2IN triggered over 50% cell death; however, when combined with either cisplatin or etoposide it increased the proportion of cells in S phase, suggesting S phase arrest. SUM1IN treatment caused substantial cell death in A549 cells, and synergized with cisplatin but not with etoposide. In HCC827 cells, SUM1IN caused 24% cell death, substantial arrest on S phase, and synergized with etoposide but not with cisplatin. Each data point represents the mean of three independent experiments. For bar graphs displaying the corresponding standard deviations, refer to Supplementary Figure 2.

Based on the effects of SUM2IN and SUM1IN on cell viability, we then examined whether these treatments also affected cell cycle progression. Unlike the cytotoxicity assay, which uses fluorescence-based viability dyes (Hoechst and Propidium Iodide) to distinguish live and dead cells via imaging, cell cycle distribution was assessed with NIM–DAPI staining using flow cytometry. This method enables a quantitative analysis of DNA content rather than membrane integrity or cell death. Following treatment, both adherent and non-adherent cells were resuspended in a permeabilizing NIM solution containing NP-40 and DAPI (NIM-DAPI). The detergent facilitates nuclear membrane permeabilization, allowing DAPI to bind stoichiometrically to DNA. Samples were then analyzed by flow cytometry to generate cell cycle profiles based on DAPI fluorescence intensity. SUM2IN treatment induced apoptosis in approximately 34% of A549 cells and 55% of HCC827 cells (Fig. 5), while SUM1IN treatment resulted in apoptosis in about 58% of A549 cells and 24% of HCC827 cells (Fig. 5). In contrast, only around 5% of apoptotic cells were detected in mock-treated and scrambled morpholino-treated A549 and HCC827 cells (Fig. 5).

Cell cycle analyses showed that SUM2IN treatment caused significant changes in the distribution of cells across different phases in both cell lines, including a decrease in the percentage of cells in G0/G1 and G2/M, along with a slight increase in the S phase population (Fig. 5). These results suggest that modifying SUMO2 impacts cell cycle progression. SUM1IN treatment resulted in a marked reduction of cells in G0/G1 and G2/M phases and a concomitant accumulation of cells in the S phase in both A549 and HCC827 cells (Fig. 5). Collectively, these data indicate that both SUM2IN and SUM1IN promote apoptosis and disrupt cell cycle progression in NSCLC cells.

Besides regulating cell cycle progression and apoptosis, SUMOylation is also known to influence cell adhesion [37], cytoskeletal structures [7], and the epithelial-to-mesenchymal transition [38], all of which are important for determining cell motility [39]. Therefore, we conducted migration assays after treating with SUM2IN and SUM1IN (Fig. 1A). These assays showed a significant decrease in migration at both 24 and 48 hours after treatment. Quantitative analyses revealed that SUM2IN reduced migration by 16% at 24 hours and 37% at 48 hours, while SUM1IN decreased migration by 54% and 57% at the respective time points (Supplementary Fig. 5), indicating a strong inhibitory effect on cell motility. Overall, these findings suggest that SUM2IN and SUM1IN inhibit NSCLC cell proliferation and migration while promoting apoptosis.

### SUM2IN and SUM1IN Sensitize NSCLC Cells to Cisplatin and Etoposide

Considering the cytotoxic and cell-cycle-inhibitory effects mediated by SUM2IN and SUM1IN described above, we evaluated whether they could enhance the anti-tumor effects of two chemotherapeutic agents commonly used to treat NSCLC: cisplatin and etoposide. We hypothesized that simultaneous inhibition of the SUMOylation system with SUM2IN or SUM1IN, combined with cisplatin or etoposide, would increase chemotherapy-induced cytotoxicity and reduce the development of chemoresistance. To assess the effectiveness of SUM2IN and SUM1IN as chemosensitizers, we tested them in combination with the aforementioned chemotherapeutic agents through cell viability, cell cycle progression, and clonogenic survival assays using A549 and HCC827 cells, with HEL299 cells serving as a non-malignant lung cell control. For all these tests, we selected concentrations of the chemotherapeutic agents around 1/5th to 1/4th of their EC50.

In the cytotoxicity assay, treatment with SUM2IN and SUM1IN significantly increased the cytotoxic effects of cisplatin and etoposide in both NSCLC cell lines (Fig. 4A, B, E, & F). Conversely, the sensitizing effects of SUM2IN and SUM1IN were notably reduced in HEL299 cells (Supplementary Fig. 1), indicating some level of tumor selectivity.

Consistent with the cytotoxicity assays, clonogenic assays showed a significant decrease in colony formation in A549 and HCC827 cells treated with SUM2IN or SUM1IN combined with cisplatin or etoposide, compared to chemotherapy alone or treatment with SUM2IN or SUM1IN alone (Fig. 4C, D, G, & H). In A549 cells, SUM2IN decreased colony formation by 30% when combined with cisplatin and by 8% when combined with etoposide, relative to treatment with the chemotherapeutic drug alone (Fig. 4C&D). In HCC827 cells, SUM2IN reduced colony numbers by 42% with cisplatin and 35% with etoposide (Fig. 4G&H). Notably, SUM1IN demonstrated greater cytotoxic and chemosensitizing effects. In A549 cells, SUM1IN lowered colony formation by 68% with cisplatin and by 54% with etoposide (Fig. 4C&D). In HCC827 cells, SUM1IN reduced colony numbers by 60% with cisplatin and 54% with etoposide (Fig. 4G&H).

Since the main effect of downregulating SUMO2 and SUMO1 seems to be inducing apoptosis, we further investigated apoptotic and cell cycle responses after using either SUM2IN or SUM1IN in combination with chemotherapeutic treatments. For SUM2IN, combining it with cisplatin in A549 cells increased cell death by 36%, while its combination with etoposide raised apoptosis by 25% (Fig. 5). In HCC827 cells, apoptosis increased by 15% with cisplatin and 24% with etoposide, along with a buildup of cells in the S phase (Fig. 5), indicating cell cycle arrest.

For SUM1IN, in A549 cells, combining it with cisplatin or etoposide increased apoptosis by 68% or 51% (Fig. 5), respectively, indicating a significant boost in chemotherapeutic cytotoxicity. Conversely, in HCC827 cells, SUM1IN did not further increase apoptosis beyond what was triggered by SUM1IN alone when combined with cisplatin (Fig. 5). However, combining SUM1IN with etoposide led to increased apoptosis and S-phase accumulation (Fig. 5). Overall, these findings show that treatment with SUM2IN and SUM1IN significantly enhances chemosensitivity in NSCLC cells, promoting apoptosis and disrupting cell cycle progression, while having limited effects on non-malignant cells.

In summary, our results show that SUM2IN and SUM1IN reduce cell viability, impair clonogenic survival, increase apoptosis, alter cell cycle progression, and decrease migratory capacity in two NSCLC cell lines. Importantly, SUM2IN and SUM1IN improve the cytotoxic effects of cisplatin and etoposide (Fig. 4) while having minimal impact on non-malignant lung cells (Supplementary Fig. 1), suggesting a preference for malignant chemosensitization. Overall, these findings establish SUMO paralog targeting as an effective strategy to slow tumor growth and boost chemotherapy response in NSCLC.

## Discussion

Despite numerous advances in chemotherapy, targeted therapies, and immune checkpoint inhibitors [40, 41], NSCLC remains a leading cause of cancer-related deaths worldwide. Resistance almost always develops, emphasizing the need for new strategies that target vital tumor survival mechanisms and improve existing treatments [42]. In this context, SUMOylation, a post-translational modification that regulates proliferation [43], stress resistance [44], and DNA repair [45], appears to be a promising target, given its hyperactivation in NSCLC and its link to poor prognosis [46].

To date, all SUMOylation inhibitors described in the literature target enzymatic components of the SUMOylation system. For instance, TAK-981 (subasumstat) [47] and COH000 [48] target the E1-activating enzyme and show strong inhibitory effects. This study presents the first inhibitors of their kind that directly target the SUMO modifiers, specifically the SUMO2 and SUMO1 paralogs, by leveraging the naturally occurring endogenous splicing variants produced in cells. Our SUMO-targeted inhibitors lead to the production of non-conjugatable SUMO protein isoforms, SUMO2α and SUMO1α. These proteins lack specific internal amino acid sequences essential for conjugation [27].

We designed SUM2IN and SUM1IN to promote exon skipping during the processing of SUMO2 and SUMO1 pre-mRNAs, respectively, thereby reducing pools of conjugatable SUMO. SUM2IN effectively shifted SUMO2 splicing toward its non-conjugatable isoform, SUMO2, significantly lowering global SUMOylation across all cell types. Conversely, SUM1IN successfully modulated splicing in A549 cells but not in HCC827 or HEL299 cells. Both inhibitors decreased cell proliferation, triggered apoptosis, and increased sensitivity to cisplatin and etoposide. These effects were more prominent in NSCLC cells than in non-cancerous lung cells (HEL299), suggesting tumor selectivity likely linked to the higher baseline activity of the SUMOylation system in cancer cells.

Vivo-morpholinos, such as those included in the formulation of SUM2IN and SUM1IN, are designed for efficient cellular uptake without the need for transfection reagents, enabling strong modulation of pre-mRNA splicing [49]. Their mechanism of action mirrors that of FDA-approved exon-skipping therapies successfully used for Duchenne’s muscular dystrophy, where oligonucleotide-driven exon removal restores functional protein production [50]. In our system, exon skipping results in the opposite outcome, disabling SUMO conjugation capacity. We observed apoptosis and cell cycle arrest, likely due to disrupted stress responses and DNA repair when SUMOylation is reduced. Notably, while both inhibitors decreased cell viability, SUM2IN primarily affected SUMO2/3 conjugation with moderate cytotoxicity, whereas SUM1IN appeared more cytotoxic and affected SUMO conjugation in a cell line-specific manner.

This cell-type specificity may result from variations in SUMO1 transcriptional elongation rates, which affect splice site accessibility during co-transcriptional splicing [51]. Consequently, differing elongation rates for SUMO transcripts across the tested cell types could enable the S1E2Acc morpholino to effectively bind the exon 2 acceptor site in A549 cells but not in HCC827 and HEL299 cells. We observed that the SUMO1 exon 2 splicing acceptor–targeting morpholino has complementarity to four nucleotides in the upstream exon (exon 1), making it possible for it to recognize the normally spliced SUMO1 mRNA. Additionally, this sequence is located five nucleotides downstream of the ATG start codon for SUMO1. Therefore, under specific chromatin conditions that influence the transcriptional velocity of the SUMO1 transcript, S1E2Acc might not affect the splicing of SUMO1 pre-mRNAs but instead block the translation of mature SUMO1 mRNAs, likely at the initiation or early elongation stages, thus decreasing *de novo* SUMO1 synthesis. This alternative mechanism of action is mediated by antisense morpholinos targeting sequences near the translational start site.

The vivo-morpholinos used in this study include a dendritic transporter with eight guanidinium groups to promote efficient uptake of the morpholino in vitro. However, vivo-morpholinos have not been approved for use in humans, and unmodified morpholinos are inefficient because they are quickly cleared by the kidneys. Therefore, achieving efficient systemic delivery of morpholinos remains a challenge for clinical use. Incorporating SUM2IN and SUM1IN into lipid nanoparticles [52, 53] or nanovesicles [54, 55], similar to platforms used for mRNA or siRNA therapies, could improve bioavailability, target specificity, and tumor accumulation *in vivo*, especially when decorated with target-specific ligands [53, 54]. These strategies could also enable combination treatments pairing splicing modulators with chemotherapeutics, taking advantage of the strong chemosensitization observed in this study.

Our findings highlight several important directions for future research. First, preclinical validation in animal models is necessary to evaluate pharmacokinetics, biodistribution, and therapeutic efficacy in vivo. Second, studies using patient-derived tumor explants and NSCLC organoids could provide translational insight into tumor heterogeneity and the variability of SUMO paralog targeting across genetic backgrounds. Third, transcriptomic profiling following SUM2IN and SUM1IN treatment will be critical to identify downstream pathways affected by SUMOylation loss, potentially revealing predictive biomarkers of response. Integrating such multiomic data with mutational profiles could enable precision-guided use of SUMO-modulating therapies, predicting chemosensitization outcomes across different cancer subtypes and drug regimens.

In summary, the data presented here demonstrate that direct modulation of SUMO paralog splicing can effectively inhibit global SUMOylation, slow tumor growth, and increase chemosensitivity in NSCLC cells. Unlike enzymatic SUMO inhibitors, SUM2IN and SUM1IN target the modifiers themselves, offering a mechanistically novel and potentially less toxic approach to suppress SUMO-dependent oncogenic signaling. This work lays the groundwork for future research into paralog-specific splicing modulation as a therapeutic strategy in cancer and possibly other SUMO-related diseases.

## Supplementary Material

Supplementary Files

This is a list of supplementary files associated with this preprint. Click to download.

• SupplementaryFiguresLandscape1.pdf

## Figures and Tables

**Table 1:** SUMO2 and SUMO1 vivo-morpholinos sequences. Vivo-morpholino pairs were designed to target the donor and acceptor splice sites surrounding the exon of interest, promoting exon skipping for further study.

Gene targeted	Inhibitor Name	Region Targeted	Vivo-morpholino name	Sequences
**SUMO1**	SUM1IN	SUMO 1 Exon 2 Acceptor	S1E2Acc	5' CTTTCAGGAGGCAAAACCTTCAACT 3'
		SUMO 1 Exon 2 Donor	S1E2Don	5' GTCATTGGACAGGTGAGTTTCATTG 3'
**SUMO2**	SUM2IN	SUMO2 Exon 3 Acceptor	S2E3Acc	5' CCTCATTGACAATCCCTGAACGAGA 3'
		SUMO2 Exon 3 Donor	S2E3Don	5' GAGATTTTTTTACCTGTGCAGGTGT 3'

**Table 2:** Primers used to evaluate the alternative splicing events driven by SUM2IN and SUM1IN. Primer pairs used to selectively amplify specific mRNA variants of SUMO1 and SUMO2. Most primers correspond to exon-exon junctions, ensuring specificity for distinct alternative splicing events.

**Primers used to evaluate SUMO2 splicing**	
S2V1V2.FW	5' ACGAAAAGCCCAAGGAAGGAGTCAAG 3'
S2V1.RV	5' TGTATCTTCATCCTCCATTTCCAACTGTGC 3'
S2V2.RV	5' TGTATCTTCATCCTCCATTTCCAACTGTCG 3'
Primers used to evaluate SUMO1 splicing	
S1V1V2.FW	5' AGAGATGGGGTGCCAGTTTTCAATTCC 3'
S1V2.RV	5' CCAGACCCTCAAATTTTAAAACTAAACTGTTGAATGACCC 3'
S1V1V3.RV	5' AGAGATGGGGTGCCAGTTTTCAATTCC 3'
S1V3.FW	5' GTCATCATGTCTGACCAGGATAGCAGTG 3'

**Table 3:** Summary of cytotoxicity data. Percentage of mock-, SUM2IN-, and SUM1IN-treated cells, either alone or in combination with cisplatin or etoposide.

A549			
	No Chemo	Cis	Eto
Mock	1.7%	28.6%	6.3%
SUM2IN	12.2%	48.4%	23.8%
SUM1IN	27.2%	48.7%	28.2%
HCC827			
	No Chemo	Cis	Eto
Mock	3.4%	20%	31.5%
SUM2IN	38.7%	51.6%	50.7%
SUM1IN	62.7%	62.8%	76.3%
HEL299			
	No Chemo	Cis	Eto
Mock	1%	47.8%	11.8%
SUM2IN	21.1%	40.4%	5.7%
SUM1IN	7.2%	71.2%	29.2%

## Data Availability

All data supporting the findings of this study are available within the paper and its Supplementary Information.
